# Analysis of Entropy Production in Structured Chemical Reactors: Optimization for Catalytic Combustion of Air Pollutants

**DOI:** 10.3390/e22091017

**Published:** 2020-09-11

**Authors:** Mateusz Korpyś, Anna Gancarczyk, Marzena Iwaniszyn, Katarzyna Sindera, Przemysław J. Jodłowski, Andrzej Kołodziej

**Affiliations:** 1 Institute of Chemical Engineering, Polish Academy of Sciences, Bałtycka 5, 44-100 Gliwice, Poland; anna.g@iich.gliwice.pl (A.G.); miwaniszyn@iich.gliwice.pl (M.I.); katarzyna.sindera@iich.gliwice.pl (K.S.); ask@iich.gliwice.pl (A.K.); 2 Faculty of Chemical Engineering and Technology, Cracow University of Technology, Warszawska 24, 31-155 Kraków, Poland; pjodlowski@pk.edu.pl; 3 Faculty of Civil Engineering and Architecture, Opole University of Technology, Katowicka 48, 45-061 Opole, Poland

**Keywords:** entropy production, optimization, reactor modelling, irreversible thermodynamics

## Abstract

Optimization of structured reactors is not without some difficulties due to highly random economic issues. In this study, an entropic approach to optimization is proposed. The model of entropy production in a structured catalytic reactor is introduced and discussed. Entropy production due to flow friction, heat and mass transfer and chemical reaction is derived and referred to the process yield. The entropic optimization criterion is applied for the case of catalytic combustion of methane. Several variants of catalytic supports are considered including wire gauzes, classic (long-channel) and short-channel monoliths, packed bed and solid foam. The proposed entropic criterion may indicate technically rational solutions of a reactor process that is as close as possible to the equilibrium, taking into account all the process phenomena such as heat and mass transfer, flow friction and chemical reaction.

## 1. Introduction

At the industrial level, optimization of chemical processes, including those based on structured catalytic reactors, is an inherent issue of the design procedure. Process optimization considers the prices of raw materials, energy, products and installations (apparatus); the prices may change rapidly and unpredictably due to market fluctuations, even at the negotiation stage. Therefore, process optimization is usually regarded as being within the engineering domain, it is in fact more connected with business and economic issues. These issues usually exceed the knowledge of an engineer or a scientist and require input from other individuals.

Structured reactors are very important in chemistry and catalysis [1,2,3]. The process design, i.e., the apparatus and the process conditions, has to secure some economic profitability in spite of potential changes of costs. Regardless of possible economic fluctuations (excluding any collapses), the process has to be profitable during the following years.

A review of the literature provides hints about recommended flow velocities, process temperatures and catalyst carriers. The data originate from the long-standing technical and economic experience of engineers and entrepreneurs. Recently, a new generation of structured catalytic reactors has been introduced into industry, and there is a paucity of knowledge and experience about their optimization. Moreover, the inner-structure design of the reactors is complicated because many geometrical parameters need to be optimized.

In the literature, different criteria can be found, which help identify optimal operating conditions of chemical reactors. “The technical” or “engineering” optimization, with which this work deals, focuses on reactor optimization in terms of fluid velocities, process (reaction) temperature, structured catalyst carrier shape and dimensions. This kind of optimization has begun in energetics due to the introduction of compact heat exchangers that usually exploit a combination of fins, turbulence mixers and other features. In the current literature, even more sophisticated criteria are proposed for multiparameter optimization of different equipment such as heat exchangers. So far, similar criteria for catalytic reactors have been derived. The comprehensive performance evaluation criteria (PEC) use three components: transport coefficients, reaction kinetics and pressure drop [4,5]. Another approach is the comparison of reactor length (or catalyst mass) with the resulting flow resistance as shown in [4,6]. For heat exchanger optimization, there are also evaluation criteria based on entropy production during the process, as presented, e.g., by London [7] and Bejan [8], who also predicted the extension of entropic criteria to chemical reactors. Entropy in economic analysis is treated as trade-off factor and can be a substitute of currency [9]. The application of entropic criterion can also be found in [10,11,12].

The aim of the study is to propose a highly simplified approach, based on irreversible thermodynamics, suitable for engineering optimization of chemical reactors. The entropic criterion is proposed to optimize structured catalytic reactors. The assumed model process is the catalytic combustion of methane.

## 2. Theoretical Background

To derive the equations governing entropy production, the reactor model must be specified. For the purposes of this paper, the one-dimensional plug-flow model (neglecting axial dispersion) in the steady-state was assumed. Due to the very thin catalyst layer deposited on the structured carrier, the internal diffusional resistance can be neglected.

Mass balance of reactant A, in the flowing fluid, per unit surface area of the reactor cross-section, is as follows:

(1) $$w_{0}\frac{{\mathrm{dC}}_{A}}{\mathrm{dx}}{+k}_{C}S_{v}\left(C_{A}-C_{\mathrm{AS}}\right)\;=\;0$$

The initial conditions are: (i) *x* = 0; *C_A_ = C_A_*_0_ and (ii) the reactant A, mass transferred from the gas bulk to the catalyst surface is balanced by the first-order catalytic reaction:

(2) $$k_{C}\left(C_{A}-C_{\mathrm{AS}}\right){\;=\;k}_{r}C_{\mathrm{AS}}.$$

Deriving concentration of A, at the catalyst surface from Equation (2), Equation (1) becomes:

(3) $$-w_{0}\frac{{\mathrm{dC}}_{A}}{\mathrm{dx}}{\;=\;S}_{v}\frac{k_{C}k_{r}}{k_{C}{+k}_{r}}C_{A},$$

and, after integration, local concentration *C_Ax_* and the reactor length *L*, required for the outlet concentration *C_AL_* are:

(4) $$C_{\mathrm{Ax}}{\;=\;C}_{A0}\exp\left(-\frac{x}{w_{0}}\frac{S_{v}k_{C}k_{r}}{k_{C}{+k}_{r}}\right),$$

(5) $$L_{}=\;\frac{w_{0}}{S_{v}}\frac{k_{C}{+k}_{r}}{k_{C}k_{r}}\ln\left(\frac{C_{A0}}{C_{AL}}\right).$$

The energy balance may be presented (assuming no heat losses to the environment) as:

(6) $$w_{0}\varrho c_{p}\frac{\mathrm{dT}}{\mathrm{dx}}{+\alpha S}_{v}\left(T-T_{S}\right)\;=\;0,$$

the initial conditions: at *x* = 0, *T* = *T*_0_.

The mass and heat transfer in a heterogeneous catalytic reactor are strictly bound up (released reaction heat depends on the reactants mass transferred to the catalyst), thus

(7) $$q_{}=\;\alpha \left(T_{S}-T\right){\;=\;-\Delta H}_{R}J_{A}{=\;-\Delta H}_{R}k_{C}\left(C_{A}-C_{\mathrm{AS}}\right).$$

The above equations assume an isothermal process. In reality, the process is adiabatic. However, the concentration of organic air pollutants is usually low. For the volatile organic compounds (VOCs), a concentration of very few ppm is typical; for methane, it depends on the kind of source and may be within 1–1000 ppm. The level of concentrations of 100 ppm and higher can be treated by homogeneous combustion in, e.g., reverse-flow reactors due to important reaction heat. Thus, we assumed the concentration of methane at 200 ppm as rational for our analysis. In such a case, the adiabatic temperature rise is about 6 K, so the temperature increase along the reactor can be securely neglected.

Entropy production is an increase of system entropy due only to the irreversible phenomena [13]. This means that there is no entropy production at equilibrium or during a quasi-static process that runs infinitely close to the equilibrium. Any industrial process runs far from the equilibrium, and it produces entropy at irreversible conditions. In irreversible thermodynamics, entropy production is derived as the product of flux *J_i_* and the driving force Δ*π* (causing the stream) divided by absolute temperature *T* [13,14]:

(8) $$S_{i}\;=\;\frac{J_{i}\Delta \pi }{T}.$$

Assuming that the stream *J_i_* is proportional to the driving force:

(9) $$J_{i}{\;=\;k}_{i}\Delta \pi ,$$

entropy production is proportional to the square of the driving force, thus it increases rapidly with the distance from the equilibrium:

(10) $$S_{i}\;=\;\frac{k_{i}{\left(\Delta \pi \right)}^{2}}{T}.$$

In this paper, entropy production is considered due to the following irreversible phenomena:

- heat transfer between the gas phase and the catalyst surface (further denoted as *H*);
- diffusional mass transfer between the gas phase and the catalyst surface (denoted as *D*);
- irreversible catalytic reaction (denoted as *R*);
- flow friction, i.e., work performed against the flow resistance (denoted as *F*).

Total entropy production (per 1 mole of reactant A consumed in the reactor) is the sum of all the components:

(11) $$S_{P}{\;=\;S}_{H}{+S}_{D}{+S}_{R}{+S}_{F}.$$

The above-mentioned components of entropy production are gathered in Table 1.

In the first column, basic equations of local entropy production are presented. In the second and third columns, the equations for the stream and the driving force are presented, respectively, derived using the reactor model. The last column presents reactor-integrated entropy production per 1 mole of substrate A consumed (e.g., burned) in the reactor. Detailed derivations, simple in fact, are not presented for reason of conciseness. The last position in Table 1, *flow friction* needs further comment. The entropy source considered is the volume fluid flow. The stream (flux) is the flow velocity and the driving force is the pressure gradient. The entropy produced is tantamount to viscous dissipation of pumping energy. This approach seems more friendly for engineers than viscous momentum flux often presented by irreversible thermodynamics; the flux is the pressure tensor and the driving force is the velocity gradient [11].

**Figure 1:**
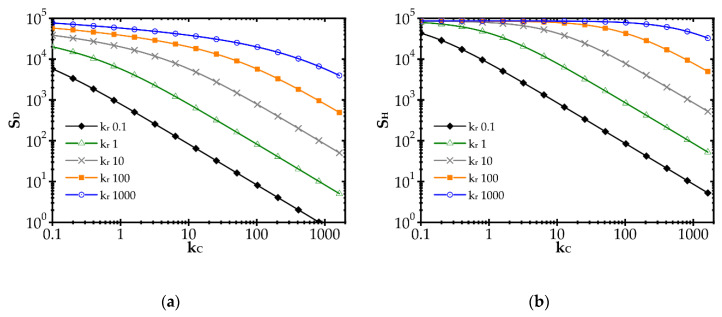
Impact of the mass transfer coefficient and reaction rate on entropy production due to: (**a**) mass transfer and (**b**) heat transfer.

The impact of the reaction rate constant, *k_r_*, and the heat and mass transfer coefficients, *α* and *k_C_*, respectively, on the entropy produced by the heat (*S_H_*) and mass (*S_D_*) transfer is illustrated in Figure 1 for the combustion process and exemplary *k_r_* and *k_C_* values. The heat and mass transfer coefficients are bound by the Chilton–Colburn analogy [15], Equation (12), which allows the influence of mass transport on *S_H_* to be determined.

(12) $$j\;=\;\frac{\mathrm{Nu}}{{\mathrm{RePr}}^{1/3}}\;=\;\frac{\mathrm{Sh}}{{\mathrm{ReSc}}^{1/3}}.$$

(13) $$S_{D}\;=\;\mathrm{Rln}\left(1+\frac{k_{r}}{k_{C}}\right),$$

(14) $$S_{H}\;=\;\frac{k_{r}}{k_{C}{+k}_{r}}\left[\frac{{\left(-∆H_{R}\right)}^{2}\left(C_{A0}-C_{\mathrm{AL}}\right)D_{A}{\mathrm{Sc}}^{1/3}}{{2\lambda T}^{2}{\mathrm{Pr}}^{1/3}}\right].$$

In Figure 1, a distinct increase of entropy produced with the reaction rate constant, *k_r_*, is observed. Conversely, entropy decreases with the mass transfer coefficient, *k_C_* (due to heat, *S_H_*, and mass, *S_D_*, transfer). A rapid chemical reaction (i.e., high *k_r_*) generates intense mass transport of substrates to the catalyst surface and adequate heat transfer in the opposite direction. The faster the reaction, the further the process runs from the equilibrium. When the transfer coefficients are small compared to the reaction rate, the concentration and temperature gradients are large, and even the substrates concentration on the catalyst goes to zero. Entropy production is large, being proportional to the square of the driving force (concentration or temperature gradient, cf. Equation (10)).

The impact of the mass transfer coefficient is opposite. The higher the transfer coefficient for a given reaction rate, the lower the temperature and concentration gradients are and the closer to the equilibrium the process runs. Smaller driving forces lead to lower entropy according to Equation (10). However, when analysing the plots in Figure 1, the impact of mass transfer intensification is distinct only if *k_C_* is close to the *k_r_* value. If *k_C_* is much smaller than *k_r_*, slight transfer enhancement will give nothing as the concentration and temperature gradients are still large (zero concentration at the catalyst surface). The gradients start to decrease as the reaction and transfer become comparable.

Obviously, the values of *k_r_* and especially of *k_C_* in Figure 1, may not be found in reality as the plots presented are theoretical, to illustrate the common impact of transfer and reaction rates on entropy production.

## 3. Catalyst Supports Considered

The aim of this study is to show the optimal adjustment of the catalyst carrier geometry, as well as its transfer and friction characteristics to the catalytic reaction kinetics. The catalyst performance (reaction kinetics) is treated as a model parameter only. Therefore, analysed catalyst supports were selected on the basis of similar value of specific surface area. This means that, in all considered cases, approximately, the same area was available for active layer catalyst deposition. For comparison, monolith and packed bed are also examined.

**Figure 2:**
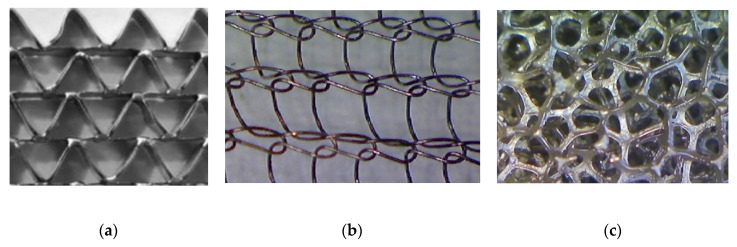
Catalyst supports: (**a**) triangular short-channel structure, (**b**) wire gauze, and (**c**) nickel chromium foam.

**Figure 3:**
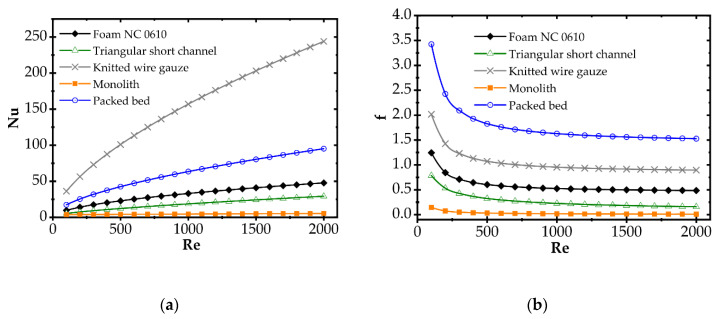
(**a**) Average Nusselt number and (**b**) Fanning friction factor for considered catalyst carriers.

Correlations for the heat transfer and Fanning friction factor were derived experimentally and presented in detail in our earlier papers [4,16]. A photo of catalyst supports considered in the study is presented in Figure 2, and a summary of equations for Fanning friction factor, Nusselt number and Sherwood number of investigated supports are presented in Table 2 and compared in Figure 3.

The kinetic tests were performed experimentally. Two different catalyst deposition methods were applied: (1) for Pd/ZrO_2_, the incipient wetness (IW) method [20] and (2) for Pd/Al_2_O_3_, sonochemical (SC) method [4]. The kinetic studies were conducted in the temperature range of 373–823 K [20]. Kinetic data are presented in Table 3. As was found in [21], the sonochemical method allows higher catalyst activity to be obtained in comparison to the incipient wetness method.

## 4. Results and Discussion

Plots referring to analysis of entropy production were constructed assuming reactor length required for 90% conversion and show the entropy produced per 1 kmole of methane combusted in the reactor under given process conditions. Entropy production is presented as a function of process temperature and the Reynolds number. Entropy is produced due to the four components denoted as *R*—reaction, *H*—heat transfer, *D*—diffusional mass transfer and *F*—flow friction. The subscript *HDFR* means total entropy produced due to the *H*, *D*, *F* and *R* components.

**Figure 4:**
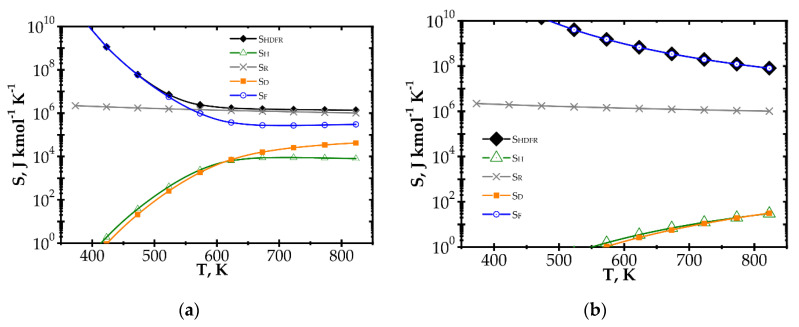
Comparison of entropy production components vs. process temperature for knitted wire gauze, Re = 1000, CH_4_ inlet concentration: 200 ppm: (**a**) fast kinetics, Pd/Al_2_O_3_ and (**b**) slow kinetics, Pd/ZrO_2_.

**Figure 5:**
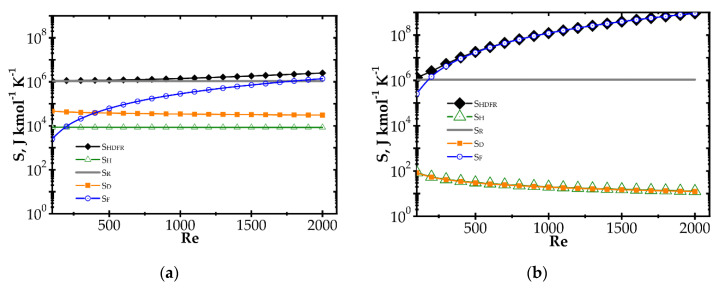
Comparison of entropy production components vs. Reynolds number for knitted wire gauze, T = 773 K, CH_4_ inlet concentration: 200 ppm: (**a**) fast kinetics, Pd/Al_2_O_3_ and (**b**) slow kinetics, Pd/ZrO_2_.

The components of entropy production (according to Table 1) for the knitted wire gauze are compared for the methane catalytic combustion process vs. process temperature (Figure 4) and the Reynolds number (Figure 5) for the fast (Pd/Al_2_O_3_) and slow (Pd/ZrO_2_) kinetics assuming initial methane concentration of 200 ppm in both cases.

When analysing the Pd/Al_2_O_3_ catalyst (Figure 4a) within the lower temperature range, entropy due to flow friction, *S_F_*, is the major component, and it is close to the total entropy production *S_HDFR_*. The heat and mass transport components, *S_H_* and *S_D_*, play less important roles. However, for higher temperatures, the kinetics become much faster, causing significant shortening of reactor length necessary to attain 90% conversion. The share of flow friction entropy decreases; simultaneously, the entropy components due to heat and mass transport play more important roles. For the highest temperature range analysed, total entropy *S_HDFR_* is close to the reaction component *S_R_,* while the remaining components are comparable. Increased entropy production due to heat and mass transport at higher temperatures is a result of faster reaction rate. This leads to lower methane concentration on the catalyst surface, and thus to higher temperature and concentration gradients, in consequence of more intense entropy production (cf. Table 1, Equation (10) and Figure 1).

For the Pd/ZrO_2_ catalyst (Figure 4b), total entropy production is close to the flow friction component in the whole temperature range analysed. The transport component *S_D_*, *S_H_* are minor due to low gradients (a result of slow kinetics), and even the reaction component *S_R_* is much lower than the flow friction one, *S_F_*.

Figure 5 illustrates entropy production as a function of the Reynolds number for knitted wire gauze assuming a rather high temperature of 773 K. The transport components *S_D_* and *S_H_* are almost constant within the whole Re range analysed. The flow friction component *S_F_* increases with Re, reaching an even higher value than *S_R_*, especially in the case of the Pd/ZrO_2_ catalyst. Moreover, in Figure 5b, the total entropy produced is close to the flow friction component, with a minor role played by the remaining components.

Large entropy production is due to the irreversible reaction of methane catalytic combustion. Moreover, this entropy component is almost the same per mole of reactant, regardless of process conditions (*T*, Re and catalyst); analysis of the equation for *S_R_* (Table 1) should render this as no surprise. Chemical affinity is close to the standard Gibbs energy of reaction (at the process temperature) Δ*G_R_^o,T^,* because the sum of the concentration logarithms is minor. For optimization purposes, the place of the minimum total entropy production reflects the process optimum, making the precise value less important. Analysis of Figure 4 and Figure 5 shows that the *S_R_* component is nearly constant within the ranges studied. Note that reaction component, *S_R_*, is the lowest possible entropy that can be produced in the chemical reactor. For engineering purposes, such as process optimization, the remaining components are more interesting because they make entropy production higher than that due to chemical reaction (*S_R_*) and they are dependent on the physical properties of carriers. For slow reaction, there is no difference between the analysed approaches, because, in this case, flow resistance plays a major role (cf. Figure 4b and Figure 5b) and the minimum is not observed within the considered temperature range. In summarising the catalytic structures displaying close specific surface area *S_v_* (i.e., similar catalyst amount), *S_R_* will be neglected during next analysis.

**Figure 6:**
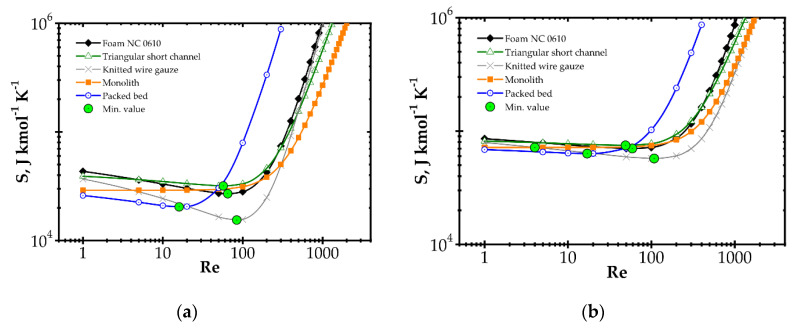
Entropy production vs. Reynolds number for different catalyst supports for the fast kinetics, Pd/Al_2_O_3_ at temperature: (**a**) 573 K and (**b**) 773 K.

**Figure 7:**
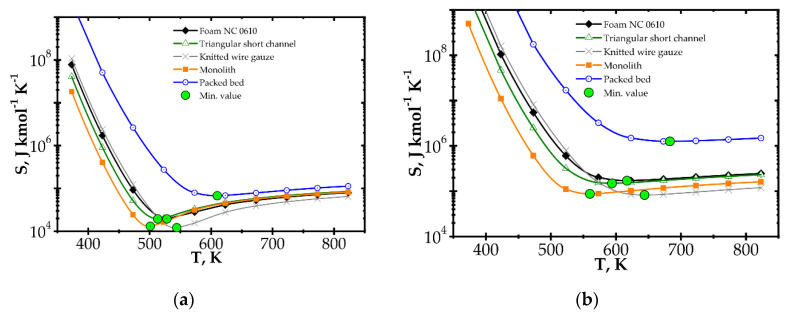
Entropy production vs. temperature for the fast kinetics, Pd/Al_2_O_3_ for different catalyst supports at Reynolds number: (**a**) Re = 100 and (**b**) Re = 500.

Analysis of entropy production due to the heat and mass transfer and flow friction (denoted as *S_HDF_*) is presented in Figure 6 and Figure 7 presents *S_HDF_* as a function of the Reynolds number and process temperature for the five catalyst supports considered. In the following figures, minimal entropy production for each support is shown; these points give optimal process conditions for particular catalyst supports.

In Figure 6, entropy is presented for two selected temperatures, moderate (573 K) and high (773 K). For the moderate temperature of 573 K (Figure 6a), packed bed seems the best for Re < 20. For Re < 500, knitted wire gauze is optimal (minimum value at Re = 84) in that this results in the lowest entropy production and the most profitable behaviour within this analysis. For a higher Reynolds number, monolith displays the lowest entropy production, undoubtedly due to its lowest flow resistance. For higher temperatures of 773 K (Figure 6b), the impact of transfer properties is more pronounced as a result of faster reaction rate, and knitted wire gauze appears to be the best with classic and short-channel monoliths. Packed bed produces the largest entropy in almost the entire Reynolds range, due to the highest flow resistance. For the higher temperature (773 K), the minima are generally slightly shifted to higher Reynolds numbers and entropy production is several times higher.

When considering temperature influence on entropy production (Figure 7), the same conclusions may be derived. Low process temperature is favourable for the classic monolith, while for higher temperatures, wire gauze and monolith seem to be the best choice. For Re = 100 (Figure 7a), above 650 K, all the internals display close entropy production. Interestingly, all the structures except packed bed show minima within the narrow range of 500–540 K. For Re = 500 (Figure 7b), entropy produced is higher, especially for packed bed. The minima are shifted towards higher temperatures by 60–100 K. Above 600 K, knitted gauze and monolith are the best.

**Figure 8:**
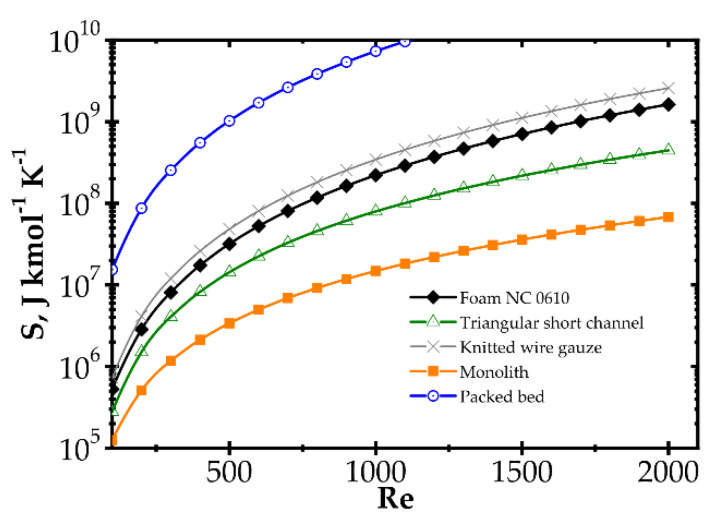
Entropy production vs. Reynolds number for different catalyst supports for the slow kinetics, Pd/ZrO_2_ at temperature 673 K.

**Figure 9:**
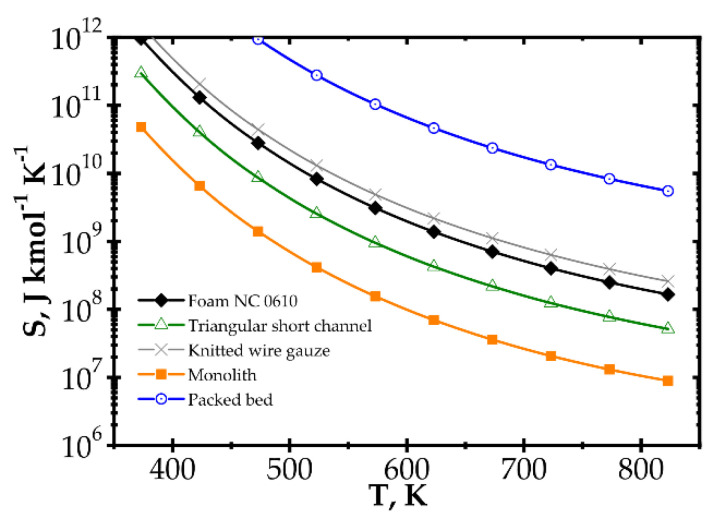
Entropy production vs. temperature for the slow kinetics, Pd/ZrO_2_ for different catalyst supports at Reynolds number 1500.

Analogous plots for slow kinetics (Figure 8 and Figure 9) show quite different behaviour. Here, the reactor is long due to the slow reaction rate. Moreover, slow reaction does not require intense heat and mass transfer. Concentration and temperature differences between the flowing fluid and catalyst surface are very small; entropy production due to transfer is small compared to that due to flow friction. Consequently, entropy produced for the slow kinetics is ordered identically to the friction factors (Figure 3b) vs. the Reynolds number and process temperature. Flow friction is the main entropy source (when neglecting chemical reaction). For slow kinetics, entropy production characteristic considered for all the internals is similar. The shift observed (towards higher or lower entropy produced) results mainly from the flow resistance. All the curves are nearly parallel, and only slight convergence is observed for low Re as a result of different transport properties. The internals displaying the lowest flow resistance (monolith and short-channel structure, cf. Figure 3) offer the lowest entropy production, while those of high flow resistance (packed bed, cf. Figure 3) produce larger entropy, so are less profitable.

## 5. Conclusions

The results obtained by entropy analysis indicate that wire gauze is the best choice for the Pd/Al_2_O_3_ catalyst and the packed bed is the worst one. In the case of the Pd/ZrO_2_ catalyst, the best carriers are monolith and short-channel structures, while the worst solution is a packed bed. However, meeting the efficiency criteria cannot be regarded as the ultimate verdict. Any process has its own characteristics and limitations. It is rare for a process to occur separately, as it is usually part of a larger installation. For example, process temperature is limited by catalyst thermal deactivation, and the flow resistance may be limited by the gas pressure available. Therefore, each process needs to be considered individually, and any overall limiting parameters must also be taken into consideration during optimization.

The entropy-based optimization methodology is able to optimize reactor structure (indicating the best geometry, specific surface, etc.), as well as the process temperature and fluid velocity for considered reaction kinetics. The criterion, ensuring the minimum entropy production*,* ignores the reactor cost and is able to indicate the best structure from among the considered ones, as well as the optimal working conditions of a reactor (e.g., temperature and flow velocity).

Irreversible chemical reaction produces almost the same entropy, per mole of reactant, regardless of the process conditions. Therefore, it can be safely neglected during entropic optimization. The hypothesis is confirmed by analysis presented in Figure 4. For proper results, entropy produced by heat transfer, mass transfer and flow friction should be accounted for.

The gauze structures are assessed as being very effective due to their satisfactory transfer and friction properties. The monolith and short triangular channel display good efficiency for slow kinetics (Pd/ZrO_2_ catalyst) due to their low flow resistance. The packed bed usually appears as an unsatisfactory solution.

For fast kinetics (Pd/Al_2_O_3_ catalyst), the transfer properties of the catalyst support are the most important for low entropy production. The intense transfer properties of, e.g., knitted wire gauze, make the support excellent for such processes. The impact of flow resistance is minor as, for a fast reaction not hampered by insufficient transfer rate, the reactor is very short.

For slow kinetics (Pd/ZrO_2_ catalyst), the reactor is long. The impact of flow resistance becomes important. In contrast, heat and mass transfer contributions to entropy production are minor. Heat and mass transfer resistance is low, so temperatures (concentrations) gradients between fluid and catalyst surface are low, and the process runs near to the equilibrium.

The optimization methodology presented in this study obviously requires further development, including thorough experimental industrial and economic application. In spite of this, the entropic criterion seems able to indicate technically rational solutions of the reactor process considering the heat and mass transfer, flow resistance and reaction kinetics.

## Figures and Tables

**Table 1:** Local and reactor-averaged components of entropy produced.

Entropy, *σ_i_*	Flux, *J_i_*	Driving Force, Δ*π*	Entropy, Reactor Average Value, *S_i_* (per mol of Substrate A)
Heat transfer (*H*) $\sigma_{H}\;=\;-\frac{q}{T^{2}}\nabla T$	Heat flux $q\;=\;-∆H_{R}J_{A}=$ $=\;\alpha \left(T_{s}-T\right)$	Temperature gradient $\left(T_{s}-T\right)\;=$ $=\;\frac{k_{C}\left(-\Delta H_{R}\right)\left(C_{A}-C_{\mathrm{AS}}\right)}{\alpha }$	$S_{H}\;=\;\frac{k_{C}k_{r}}{k_{C}{+k}_{r}}$ $\frac{{\left(-\Delta H_{R}\right)}^{2}\left(C_{A0}-C_{\mathrm{AL}}\right)}{{2\alpha T}^{2}}$
Mass transfer (*D*) $\sigma_{D}\;=\;-\sum_{i}\frac{J_{i}}{T}\nabla \mu_{i}$	Diffusive mass flux $J_{A}{\;=\;k}_{C}\left(C_{A}-C_{\mathrm{AS}}\right)\;=\;$ ${=\;k}_{\mathrm{Cr}}C_{A}$	Chemical potential gradient $\nabla \mu_{A}\;=\;\mathrm{RT}\frac{\mu_{A}-\mu_{\mathrm{AS}}}{s_{\mathrm{ef}}}$	$S_{D}=\mathrm{Rln}\left(\frac{k_{C}{+k}_{r}}{k_{C}}\right)$
Reaction (*R*) $\sigma_{R}\;=\;-\frac{{\mathrm{Ar}}_{A}S_{v}}{T}$	Reaction rate $r_{A}{\;=\;k}_{r}C_{\mathrm{AS}}{\;=\;k}_{\mathrm{Cr}}C_{A}$	Chemical affinity $A_{}=\;-\;\sum_{i}\nu_{i}\mu_{i}\;=$ $=\;-∆G_{R}^{o,T}-\;\mathrm{RT}\sum_{i}\nu_{i}{\mathrm{lny}}_{i}$	$S_{R}\;=\;\frac{A}{T}$
Flow friction (*F*) $\sigma_{F\;}=\;\frac{W}{{\mathrm{TF}}_{c}L\;}=\;-\frac{w\nabla P}{T}$	Fluid stream w	Pressure gradient −∇P	$S_{F}=\frac{f}{2T}\frac{w_{0}^{3}\varrho }{\varepsilon^{3}k_{\mathrm{Cr}}}\frac{\ln\left(\frac{C_{A0}}{C_{\mathrm{AL}}}\right)}{{(C}_{A0}{-C}_{\mathrm{AL}})}$

**Table 2:** Correlations used to calculate flow resistance, heat and mass transfer for analysed catalyst supports.

Structure Description	Correlations
Wire gauze [4]	f = 118.09/Re+0.836 Nu = 2.19Re0.636Pr1/3 Sh = 2.19Re0.636Sc1/3 $S_{v}\;=\;1355$ ε = 0.97
Triangular short channel [16]	$\left(\mathrm{fRe}\right)\;=\;13.33+11.59{\left(L^{+}\right)}^{-0.514}$ $\mathrm{Nu}\;=\;(3.11+0.45{\left(L^{*}\right)}^{-0.61})(0.55{\left({\mathrm{PrL}}^{*}\right)}^{-0.15})$ $\mathrm{Sh}\;=\;(3.11+0.45{\left(L^{*M}\right)}^{-0.61})(0.55{\left({\mathrm{PrL}}^{*M}\right)}^{-0.15})$ $S_{v}\;=\;1314$ ε = 0.95
Nickel chromium foam (NC 0610), Recemat^®^ (Dodewaard, The Netherlands); [4]	f = 79.9/Re+0.445 Nu = 0.96Re0.53Pr1/3 Sh = 0.96Re0.53Sc1/3 $S_{v}\;=\;1298$ ε = 0.89
Monolith [17]	$\left(\mathrm{fRe}\right)\;=\;14.23{\left(1+0.045{/L}^{+}\right)}^{0.5}$ $\mathrm{Nu}\;=\;3.608{\left(1+0.095{/L}^{*}\right)}^{0.45}$ $\mathrm{Sh}\;=\;3.608{\left(1+0.095{/L}^{*M}\right)}^{0.45}$ $S_{v}\;=\;1339$ ε = 0.72
Packed bed [18,19]	$f_{}=\;\frac{\left(\varepsilon -1\right)\left[600\eta \left(\varepsilon -1\right)-{7D}_{h}\varrho w\right]}{{8D}_{h}\varepsilon \varrho w}$ Nu = 2+1.1Re0.6Pr1/3 Sh = 2+1.1Re0.6Sc1/3 $S_{v}\;=\;1240$ ε = 0.38

**Table 3:** Kinetic data of tested catalysts.

Catalyst	Pre-Exponential Coefficient in Arrhenius Equation, *k*_∞_, m s^−1^	Activation Energy, *Ea*, kJ mol^−1^
Slow kinetic, incipient wetness (IW) Pd/ZrO_2_	252.49	62.79
Fast kinetic, sonochemical (SC) Pd/Al_2_O_3_	1.07·10^10^	110.4

## Nomenclature

*A*	chemical affinity, J mol^−1^
*C_A_*	reagent concentration, mol m^−3^
*c_p_*	heat capacity, J kg^−1^ K^−1^
*D_A_*	diffusivity, m^2^ s^−1^
*D_h_*	hydraulic diameter, = 4*εS_v_*^−1^, m
*F_c_*	reactor cross-sectional area, m^2^
*f*	Fanning friction factor, = ϱ*w*_0_^2^*L*(2Δ*PD_h_ε*^2^)^−1^
*J_A_*	diffusional mass flux, mol s^−1^ m^−2^
*J_i_*	stream (flux) of irreversible process, Equation (9)
*k_C_*	mass transfer coefficient, m s^−1^
*k_r_*	kinetic rate constant of the first-order reaction, referred to the catalyst surface area, m s^−1^
*k_∞_*	pre-exponential coefficient in Arrhenius equation, m s^−1^
*k_Cr_ = k_C_k_r_/(k_C_+k_r_)*	combined transfer-reaction coefficient, m s^−1^
*L*	bed length, m
*ΔP*	pressure drop, Pa/m
*q*	heat flux, W m^−2^
*R*	gas constant, J mol^−1^ K^−1^
*r_A_*	reaction rate, mol m^−2^ s^−1^
*S*	entropy production rate, J K^−1^mol^−1^
*s_ef_*	film thickness, m
*S_v_*	specific surface area, m^2^ m^−3^
*T*	temperature, K
*W*	pumping power, W
*w_0_*	superficial fluid velocity, m s^−1^
*y_i_*	mole fraction
*ΔH_R_*	reaction enthalpy, J mol^−1^
*ΔG_R_*	reaction Gibbs energy, J mol^−1^
**Greek symbols**	
*α*	heat transfer coefficient, W m^−2^ K^−1^
*ε*	porosity
*η*	dynamic viscosity, Pa s
*λ*	thermal conductivity, W m^−1^ K^−^^1^
*µ*	chemical potential, J mol^−1^
*ν*	stoichiometric coefficient
*Δπ*	driving force of irreversible process
ϱ	density, kg m^−3^
*σ*	entropy production per m^3^ of reactor volume, W m^−3^ K^−1^
**Dimensionless numbers**	
*L^+^*	dimensionless length for the hydrodynamic entrance region, = *LD_h_*^−1^Re^−1^
*L^*^*	dimensionless length for the thermal entrance region, = *LD_h_*^−1^Re^−1^Pr^−1^
*L^*M^*	dimensionless length for the mass transfer entrance region, = *LD_h_*^−1^Re^−1^Sc^−^^1^
Pr	Prandtl number, = *ηc_p_λ*^−1^
Re	Reynolds number, = *w*0*Dh* ϱ*η*^−1^*ε*^−1^
Sc	Schmidt number, = *η* ϱ−1*D_A_*^−1^
Sh	Sherwood number, = *k_C_D_h_D_A_*^−1^
**Subscripts**	
*A*	key reactant
*D*	entropy production due to mass transfer
*F*	entropy production due to flow friction
*H*	entropy production due to heat transfer
*P*	total entropy production
*R*	entropy production due to chemical reaction
*S*	catalyst surface
*x*	reactor arbitrary axial coordinate
*0, L*	reactor inlet, outlet
